# Terrestrial laser scanning and low magnetic field digitization yield similar architectural coarse root traits for 32-year-old *Pinus ponderosa* trees

**DOI:** 10.1186/s13007-024-01229-9

**Published:** 2024-07-09

**Authors:** Antonio Montagnoli, Andrew T. Hudak, Pasi Raumonen, Bruno Lasserre, Mattia Terzaghi, Carlos A. Silva, Benjamin C. Bright, Lee A. Vierling, Bruna N. de Vasconcellos, Donato Chiatante, R. Kasten Dumroese

**Affiliations:** 1 Department of Biotechnology and Life Science, University of Insubria, Varese, Italy; 2 USDA Forest Service, Rocky Mountain Research Station, Moscow, ID USA; 3 Computing Sciences, Tampere University, Tampere, Finland; 4 Department of Biosciences and Territory, University of Molise, Pesche, Italy; 5 Department of Biosciences, Biotechnologies and Environment, University of Bari Aldo Moro, Bari, Italy; 6 School of Forest, Fisheries, and Geomatics Sciences, University of Florida, Gainesville, FL USA; 7 Department of Natural Resources and Society, University of Idaho, University Federal of Parana, Moscow, ID USA; 8 University Federal of Parana, Curitiba, Brazil

**Keywords:** AMAPmod, LiDAR, Ponderosa pine, Quantitative structure models, Root architecture, TreeQSM

## Abstract

**Background:**

Understanding how trees develop their root systems is crucial for the comprehension of how wildland and urban forest ecosystems plastically respond to disturbances such as harvest, fire, and climate change. The interplay between the endogenously determined root traits and the response to environmental stimuli results in tree adaptations to biotic and abiotic factors, influencing stability, carbon allocation, and nutrient uptake. Combining the three-dimensional structure of the root system, with root morphological trait information promotes a robust understanding of root function and adaptation plasticity. Low Magnetic Field Digitization coupled with AMAPmod (botAnique et Modelisation de l’Architecture des Plantes) software has been the best-performing method for describing root system architecture and providing reliable measurements of coarse root traits, but the pace and scale of data collection remain difficult. Instrumentation and applications related to Terrestrial Laser Scanning (TLS) have advanced appreciably, and when coupled with Quantitative Structure Models (QSM), have shown some potential toward robust measurements of tree root systems. Here we compare, we believe for the first time, these two methodologies by analyzing the root system of 32-year-old *Pinus ponderosa* trees.

**Results:**

In general, at the total root system level and by root-order class, both methods yielded comparable values for the root traits volume, length, and number. QSM for each root trait was highly sensitive to the root size (i.e., input parameter *PatchDiam*) and models were optimized when discrete *PatchDiam* ranges were specified for each trait. When examining roots in the four cardinal direction sectors, we observed differences between methodologies for length and number depending on root order but not volume.

**Conclusions:**

We believe that TLS and QSM could facilitate rapid data collection, perhaps in situ, while providing quantitative accuracy, especially at the total root system level. If more detailed measures of root system architecture are desired, a TLS method would benefit from additional scans at differing perspectives, avoiding gravitational displacement to the extent possible, while subsampling roots by hand to calibrate and validate QSM models. Despite some unresolved logistical challenges, our results suggest that future use of TLS may hold promise for quantifying tree root system architecture in a rapid, replicable manner.

## Background

Understanding rooting patterns has important implications for wildland [1, 2] and urban [3] forest management, ecosystem restoration [4], and climate change mitigation [5]. In particular, the morphological traits (i.e., volume, length, diameter, and number) of tree coarse roots (≥ 1 cm diameter) can inform researchers about plant development processes in response to biotic and abiotic factors, such as biomass and carbon allocation. When knowledge of the three-dimensional structure of the root system, referred to as root system architecture (RSA) [6–9] is combined with root morphological trait information, then we can more fully understand root function and adaptation plasticity. These combinations can include genetically determined development traits (i.e., endogenous), and also modifications in response to environmental signals (i.e., exogenous), such as mechanical stress (i.e., wind and slope effects; e.g., Danjon et al. [10–12] and foraging for water and nutrients (e.g., Rewald et al. [13–17].

For about three decades, Low Magnetic Field Digitization (LMFD) coupled with AMAPmod (botAnique et Modelisation de l’Architecture des Plantes) software (hereafter simply LMFD) has represented the exclusive methodology for describing accurate root system architecture and reliable 3-dimensional (3D) measurements of root volume, length, topology, and spatial distribution of coarse (> 1 cm diameter) roots [18]. Although LMFD has made a significant contribution to root research, the pace and scale of data collection entrusted to LMFD remain difficult, tedious, and time-consuming. In addition, the LMFD approach requires carefully transporting intact root systems to a properly established digitizing station for analysis. For example, using LMFD, some root systems used in this study required up to 16 h to be digitized.

During the past two decades, instrumentation and applications related to Terrestrial Laser Scanning (TLS) have advanced appreciably. Portable TLS units and faster computers with new data processing software are now enabling a wide frontier of applications related to quantifying aboveground tree metrics. TLS has been used to estimate tree volume [19], and Quantitative Structure Models (QSM) have emerged as the leading technique for estimating not just volume but other tree structure attributes [20–25]. In contrast to the number of studies that have used TLS to characterize aboveground tree attributes, few studies have used TLS to estimate root traits [26, 27]. Here, we describe what we believe to be a novel use of TLS and QSM (hereafter simply TLS) to depict root system architecture and to estimate root traits such as length, volume, and number by root order.

## Materials and methods

### Root collection

We selected ten, 32-year-old *Pinus ponderosa* trees growing in the University of Idaho Experimental Forest in northern Idaho USA (lat 46.842240, long − 116.871035). These trees were outplanted in 1986 as part of a greenhouse–field study that explored a copper root-pruning technique applied during container nursery production toward potentially improving seedling quality [28]. A subset of outplanted trees was previously sampled [29]. Our tree designations (i.e., C or T) refer to the trees from the original control and treatment populations, respectively; various aspects of the root systems of these trees have been reported [11, 30]; Montagnoli 2019b, 2020). Full site and excavation details are presented in Dumroese et al. [11]. Briefly, trees were felled on a slope of 32–40º with a prevailing northeast aspect. We drove a screw into the bark at the root-stem interface (i.e., collar) to record north, cut the stem near the collar, drilled four screws vertically into the stump and adjusted their heights to record the horizontal level (in perpendicular directions), and excavated the roots using a high-pressure air spade. Excavated root systems were about 1 to 1.5 m in depth (distance to bedrock) and extended about 1.5 m horizontally from the trunk. Any remaining, unexcavated roots were cut, and the root systems were carefully lifted and moved to the United States Department of Agriculture, Rocky Mountain Research Station (Moscow, ID).

### Low magnetic field digitizing (LMFD)

**Fig. 1:**
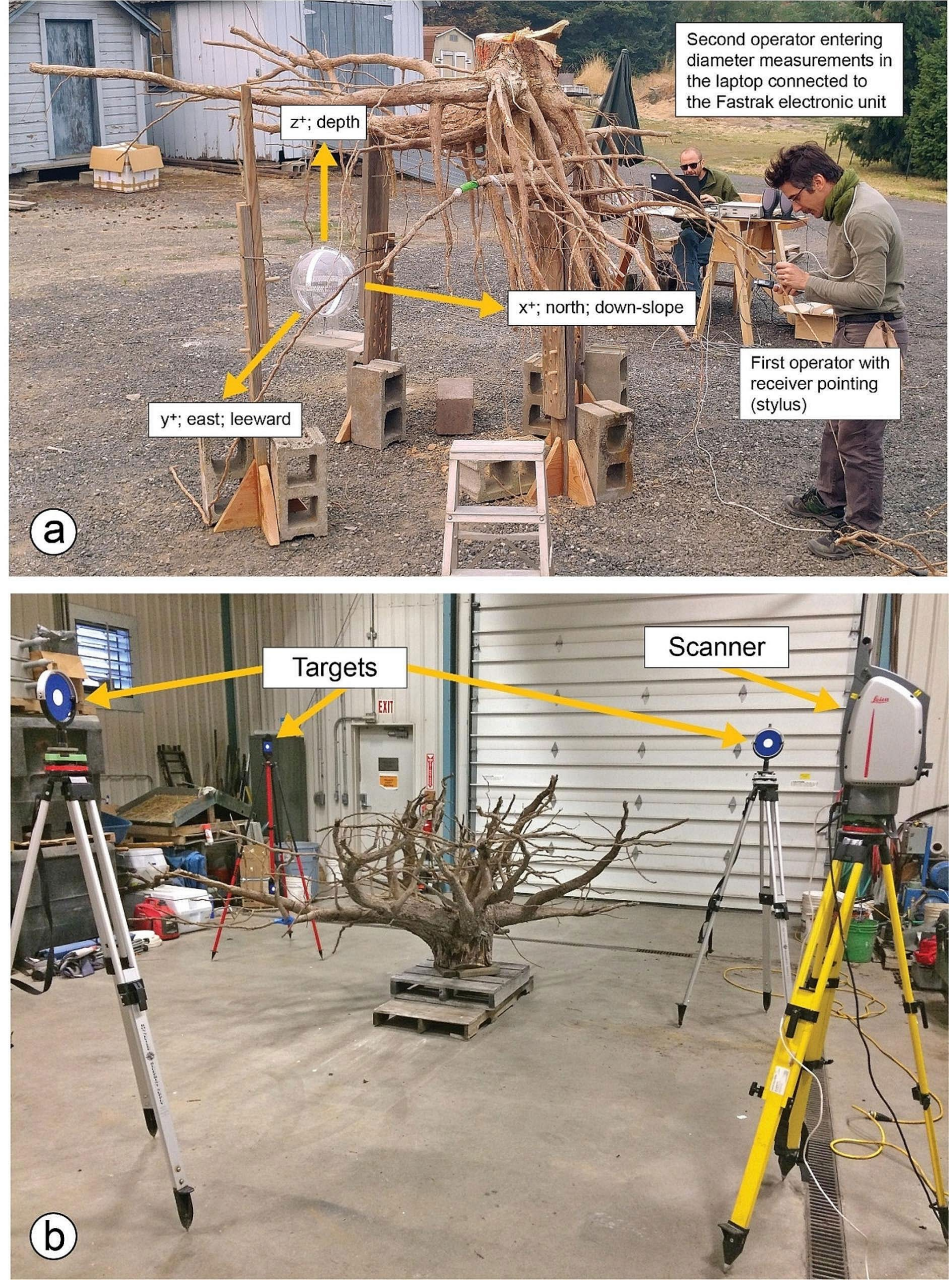
Typical arrangement of the equipment to complete Low Magnetic Field Digitization (**a**; tree T1) and Terrestrial Laser Scanning (**b**, tree T5) of a root system

**Fig. 2:**
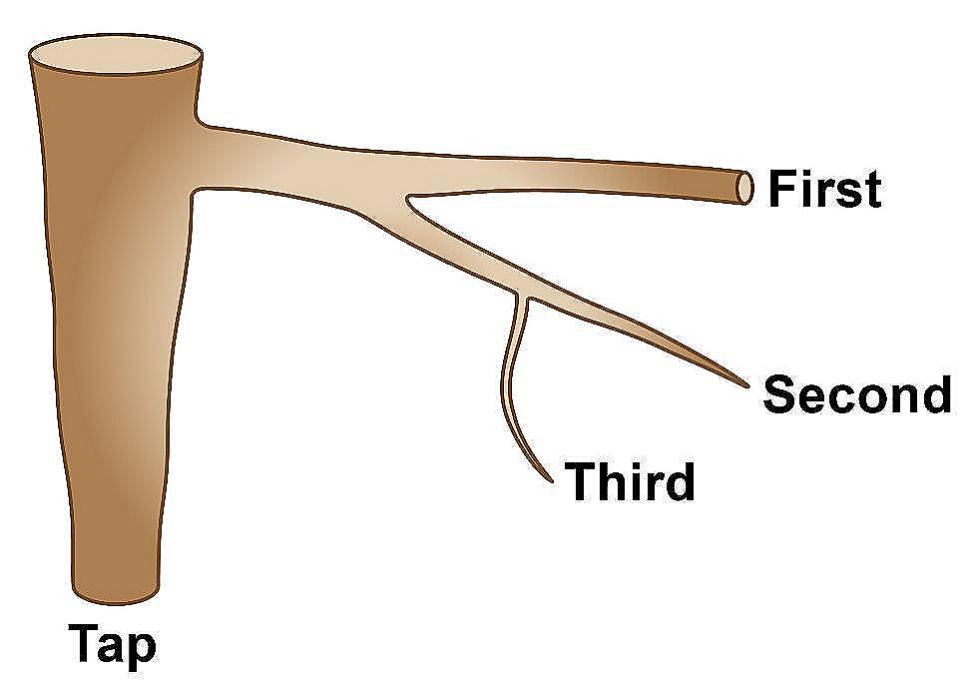
Schematic of root order hierarchy according to the centrifugal classification [34] whereby first-order roots originate at the tap, second-order roots originate at first-order roots, and so on

**Fig. 3:**
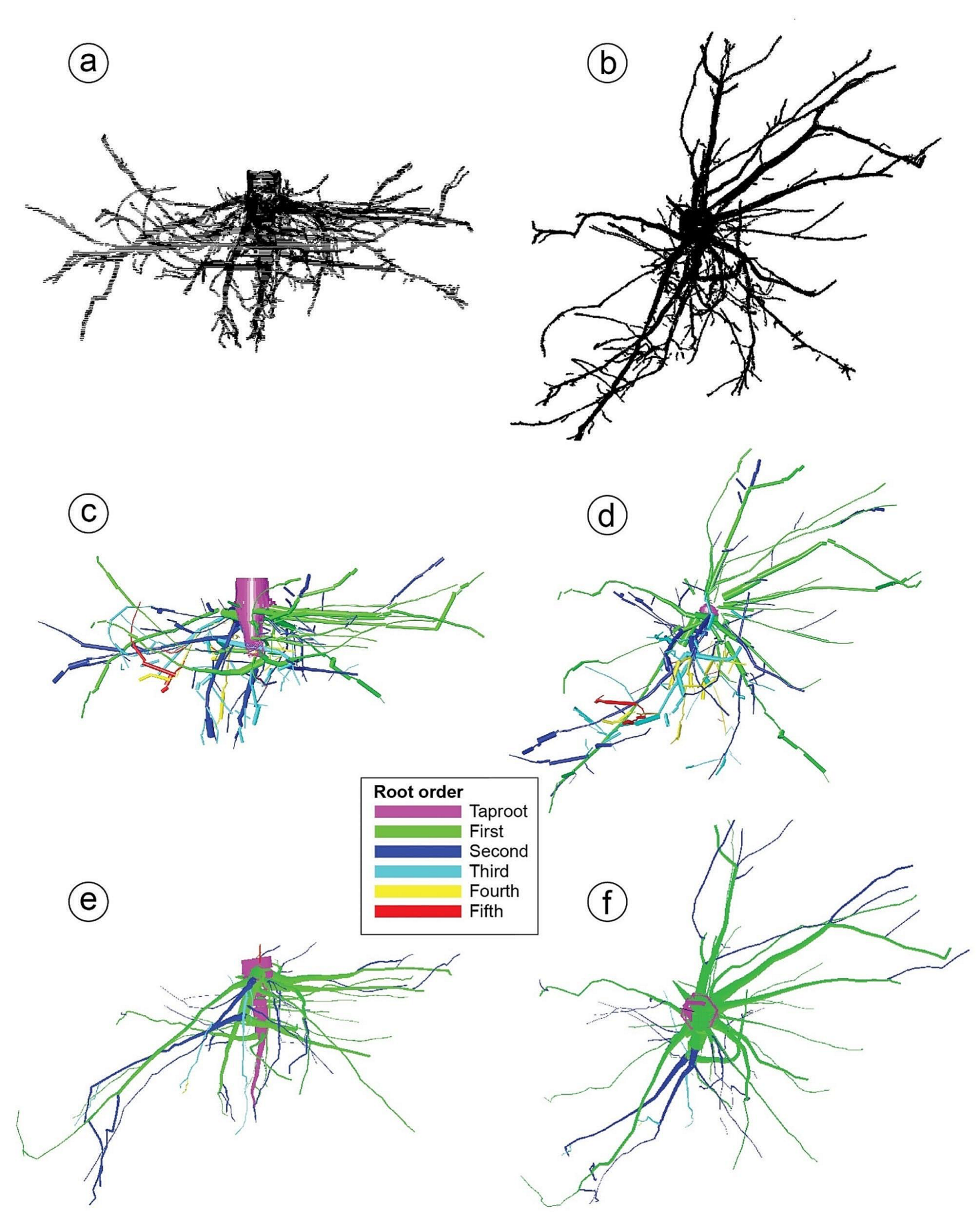
Digital reconstructions of the tree T4 root system using TLS (**a**, point cloud profile; **b**, point cloud plane; **c**, QSM profile; **d**, QSM plane) and LMFD (**e**, profile; **f**, plane)

At the laboratory, each root system was re-positioned so that the slope angle inclination (achieved by adjusting the root so that the screw heads were horizontal) and north azimuth were restored prior to characterization by LMFD to ensure accurate descriptions of root architectural traits (i.e., root spatial displacement) to mechanical constraints, such as slope and/or dominant wind. Once repositioned, we used a low magnetic field digitizer (Polhemus, Colchester, VT, USA), consisting of an electronic unit, a magnetic transmitter (Long Ranger; Polhemus), and a small hand-held receiver (Fastrak; Polhemus) to discretize each root system [18]. The transmitter was positioned approximately 1.5 m below and 2.5 m horizontally from the stump with north in the positive X direction (Fig. 1a). The transmitter generated a 4-m sphere-wide electromagnetic field in which the X, Y, and Z spatial coordinates of roots were measured with the receiver. Root topology (i.e., the branching hierarchic structure) was coded using the “acropetal-development approach” [10, 11, 31]. First-order roots emerged directly from the taproot, second-order roots from first-order roots, and so on [32]; Fig. 2). We subjectively determined the stump to be the portion of the largest vertical taproot where most of the large horizontal surface roots originated. Starting at the root collar, we followed a recursive path along the branching network [6], taking intermediate measurements between branching points to record changes in root direction and taper. These measurements were about 2 cm distant when roots were curved and approximately 15 cm when roots were straight. At each point, we measured root diameter but when a root cross-section was noticeably oblong, we recorded the largest diameter and its orientation and the diameter perpendicular to the largest diameter. We measured all roots having a proximal diameter ≥ 1 cm at their base. Data were encoded in a standard format (MTG). Output data were analyzed using the AMAPmod software [33] to provide topological structure, length, volume, and a 3D graphical reconstruction (Fig. 3a, c).

### Terrestrial laser scanning (TLS)

Root systems were scanned with a TLS (ScanStation 2, Leica Geosystems Inc., Heerbrugg, Switzerland) set to the configuration parameters shown in Table 1. Each root system was inverted, with the cut face of the stump placed on a shipping pallet as shown in Fig. 1b, to prevent the weight of the stump from compressing the 3D root distribution. The TLS was positioned to scan each root system from three 120º view positions between three reflective targets also positioned laterally at 120º from each other and within 6 m of the root system (Fig. 1b). The three targets and proprietary software (Leica Cyclone Version 7.3, Leica Geosystems Inc.) were used to merge the three separate 3D point cloud scans into a single, integrated point cloud. The XYZ text format files were exported and converted to LAS binary format files for further processing. Software (CloudCompare; open source: https://www.danielgm.net/cc/) was used to remove background objects (e.g., pallets) and noise from the point clouds.

**Table 1:** Terrestrial laser scanner (TLS) configuration parameters used

Parameter	Value(s)
Wavelength (nm)	550
Horizontal field of view (degrees)	360
Vertical field of view (degrees)	270
Scan range (m)	3–6
Maximum scan rate (s^− 1^)	50,000
Spot size (mm)	4
Beam divergence (µrad)	60
Surface precision (mm)	2

#### Root segmentation

To segment the roots using the branch-segmentation method of TreeQSM [35, 36], the entire point cloud was covered with small patches (subsets of the point cloud), with patch diameter (*PatchDiam*) selected by the user. Starting at the taproot, the base of each first-order root was determined by expanding the taproot with three layers of neighboring patches and determining the connected components of this expansion layer that could be extended further from the initial set of root bases. Because of occlusion in the data, the neighbor relation of the patches has missing connections, and the bases of some larger roots were in multiple connected components. Therefore, to improve a root base by potentially connecting multiple components into one root base, all the patches to the component, which were not yet assigned in other already defined root bases, were selected as potentially belonging to the root base. A cylinder was then fitted to the selected patches, and only those patches that were close enough to the cylinder were selected to form the final root base. The bases of the first-order roots defined above provided the subsequent bases for the branch-segmentation method of TreeQSM. The resulting segments were further cut and combined to form segments as long as possible while maintaining a length similar to the linear distance between the segment base and tip.

#### Cylinder modeling

Each root was modeled with a set of cylinders using the cylinder fitting method of TreeQSM v2.4.1. For each sub-section of the root, a series of cylinders having different lengths were evaluated, and the cylinder having the highest surface coverage of points was selected. Basically, the surface coverage of points measures how large a portion (in relative terms) of the cylinder surface is covered with TLS points. To compute this, the points were partitioned into cells based on their angles and heights as seen from the cylinder axis. If a cell had points, it was counted, and the final surface coverage was the number of non-empty cells divided by the number of all cells. Thus, each cylinder was adapted to the local curvature and point coverage of the root. This process allowed us to generate 3D visualizations of each root system (Fig. 3b, d).

#### Model optimization

In the above modeling process, the initial value of the patch diameter input parameter (i.e., *PatchDiam*) had a predominant effect on the QSM output. To standardize selection of the *PatchDiam* values, we first reconstructed QSMs across a range of 18 patch diameters (i.e., *PatchDiam =* [5, 7.5, 10, 12.5, 15, 17.5, 20, 25, 30, 35, 40, 45, 50, 60, 70, 80, 90, 100] mm).

The point density was estimated by covering the point clouds with small patches, projecting each patch to a plane defined by the two largest principal components of the patch, defining the area of the projected patch with a convex hull. Finally, the point density of the patch was considered as the number of points divided by the area. The median value of the TLS point spacing was 1.6 mm.

**Fig. 4:**
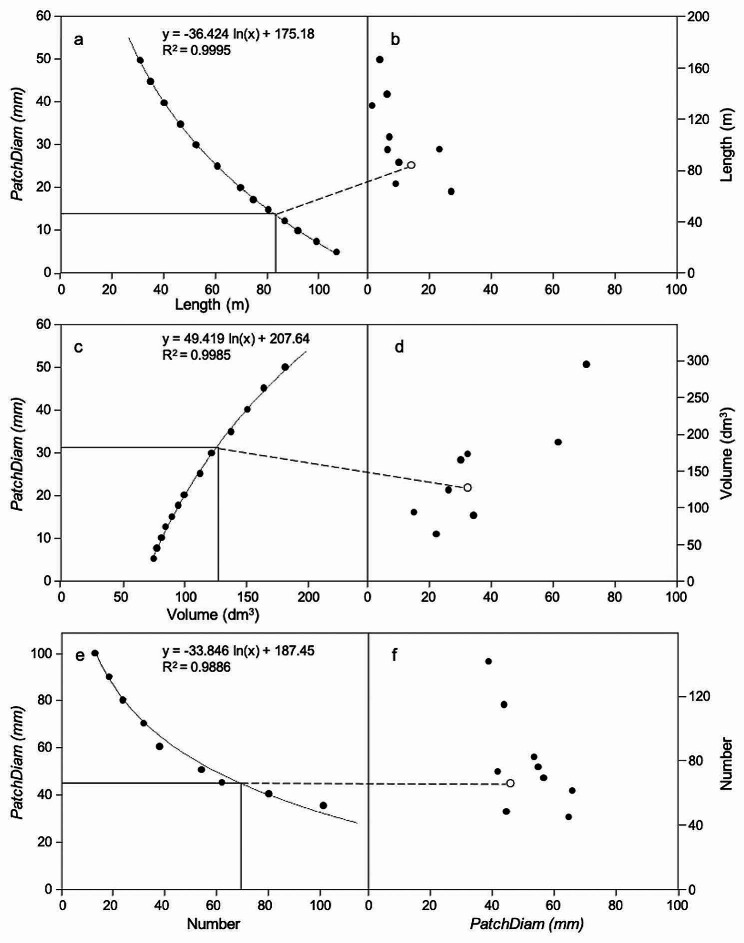
Fitted interpolation lines of predicted total root length (**a**), volume (**c**), and number (**e**) values for the ranges of *PatchDiam* and the trait value for tree C1 determined using AMAPmod (optimum length = 13.8, volume = 31.8, number = 46). Optimum *PatchDiam* for all ten trees for length (**b**), volume (**d**), and number (**f**) with tree C1 shown as white circles

For each root system and root trait (root length, volume, number), we plotted the predicted value for each *PatchDiam* and fitted an interpolation line (all R^2^ ≥ 0.97) using linear regression through the Straight method (SPSS 25.0, SPSS Inc., Chicago, IL, USA). For each root system and trait combination, we then intersected each fitted interpolated line with the trait value determined using AMAPmod (Fig. 4a, c, e). This yielded a single set of ten optimum *PatchDiam* values (one per root system) for predictive purposes, treating each root system as a sample representative of the population with equal weight (Fig. 4b, d, f). Then, we optimize the inputs with brute force using 5 models per input and used these optimum values to make 20 models per root system × trait combination to consider the randomness in the surface coverage generated and its effects on the segmentation and root trait (i.e., length, volume, and number; each trait by root order and total) modeling. The 20 models per patch diameter allowed us to estimate the variability and uncertainty of the results and quantify their precision.

Because the location of root traits (length, volume, number) is as important as their total value, we calculated root trait values in four quadrants (north, east, south, west) and two depths (< 30 cm and > 30 cm). These root displacement data help explain the plastic response of roots to mechanical forces, such as slope and dominant wind direction. For our trees, north corresponded with downslope direction and west corresponded to dominant wind direction.

#### Model results summarization

For each root system, we summarized root traits (i.e., length, volume, number) from the 20 models generated for each of the interpolated patch diameter values using the median (50th ) and 5th and 95th percentile statistics. We regressed the observed LMFD trait value on the means of the predicted TLS trait values, and calculated the variance explained (R^2^) by the best fit linear regression and its significance (*p*-value), to statistically assess agreement between the two methods.

## Results

**Fig. 5:**
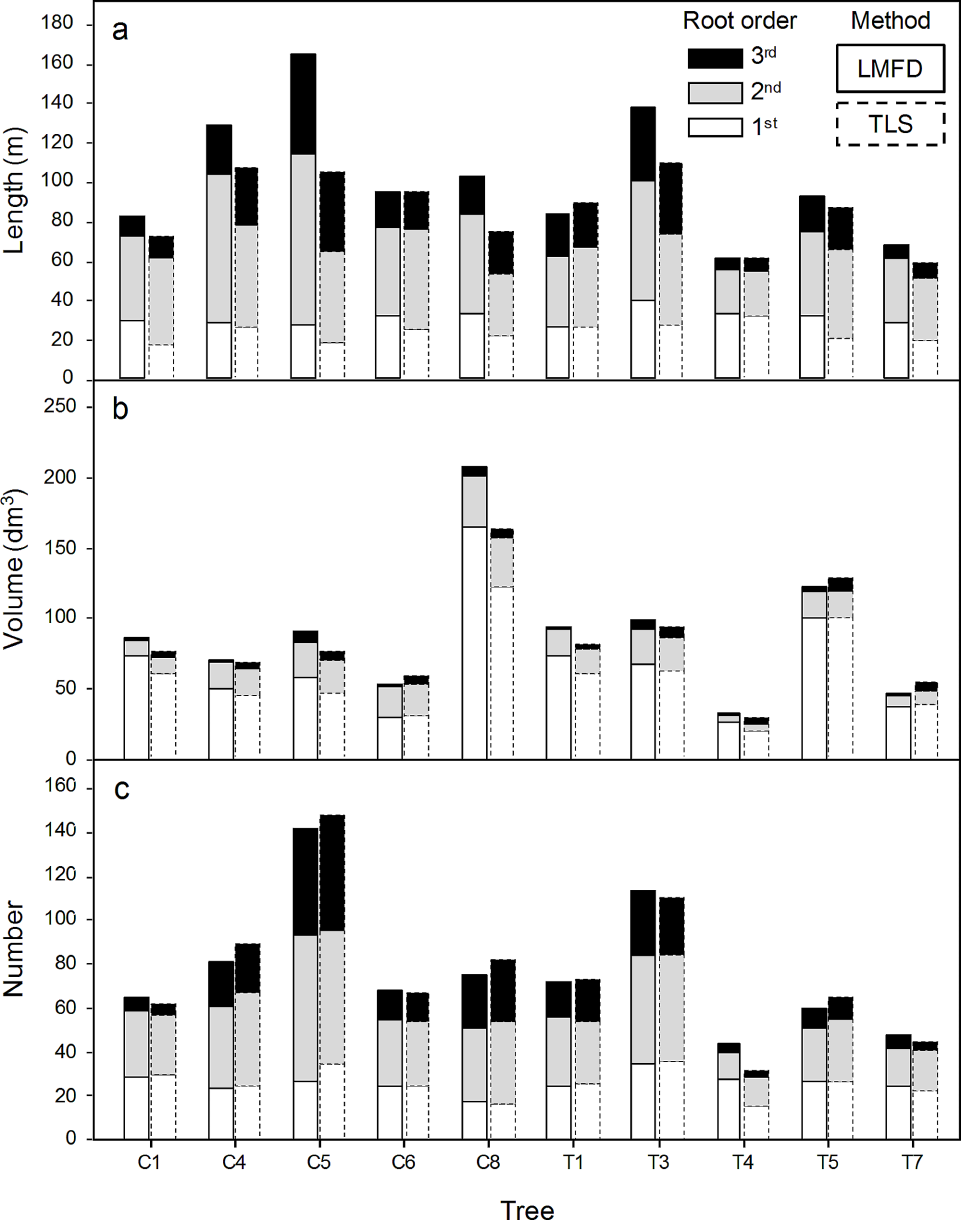
Length (**a**), volume (**b**), and number (**c**) of roots for ten root systems from control (**C**) and treatment (**T**) populations were determined using LMFD (bars with solid outline) and TLS (bars with dashed outline). Contributions to the total for each trait are shown by first- (white), second- (gray), and third (black) order roots

For the ten trees, the total (median) root volumes and lengths determined using LMFD ranged from 32.1 to 207.5 (90.3) liters and 61.2–164.2 (101.5) meters, respectively (Fig. 5). The range (median) of total root number (all classes combined) was 44–141 (76.7), with the median number of first-, second-, and third-order roots being 26.0, 33.2, and 17.5, respectively (Fig. 5). For the ten trees, the total (median) root volumes and lengths determined using TLS ranged from 28.8 to 163.9 (83.0) liters and 51.5–96.7 (75.4) meters, respectively (Fig. 5). The range (median) of total root number (all classes combined) was 31–147 (77), with the median number of first-, second-, and third-order roots being 25.2, 33.6, and 18.2, respectively (Fig. 5).

### Patch diameter optimization

TreeQSM was highly sensitive to the input parameter *PatchDiam* for each root trait. We noted that the model was optimized when discrete *PatchDiam* ranges were used but the optimal *PatchDiam* differed for each root trait (Fig. 4). Total length decreased as *PatchDiam* increased from zero to 25 mm whereas total volume increased as *PatchDiam* increased from 15 to 40 mm. The largest *PatchDiam* values (35–65 mm) yielded the best estimates of total root number.

### Comparing LMFD and TLS

**Fig. 6:**
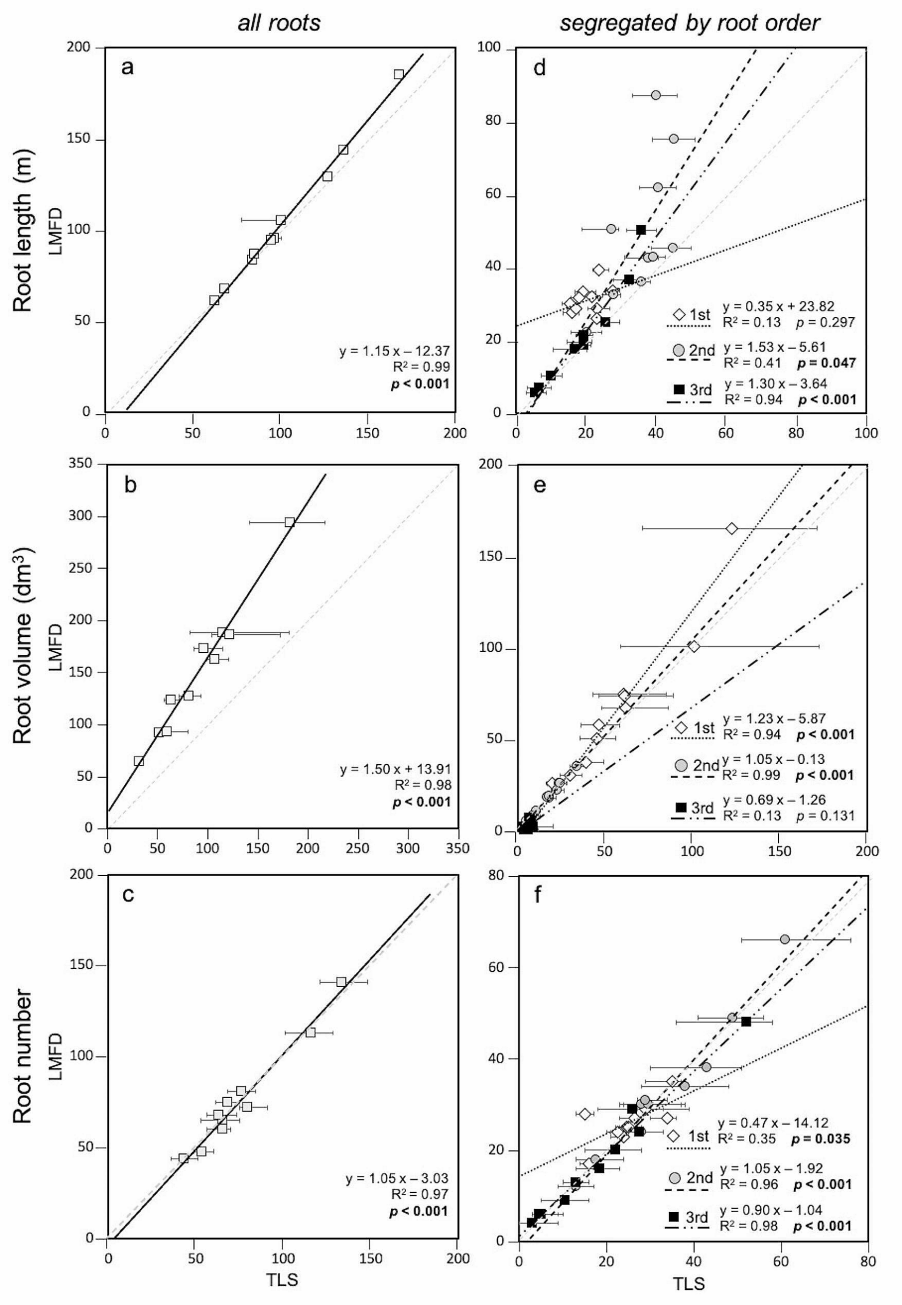
The relationship between root length (m), volume (dm^3^), and number (columns) for all roots pooled together and divided into the three root orders (rows) obtained with LMFD and TLS. The dashed light-gray line represents the 1:1 relationship

For all root systems, regression analyses of total volume, length, and number of roots ≥ 1 cm diameter were significant (all *p* ≤ 0.001; Fig. 6a, b, c). When root order was considered, regression analyses remained significant (all *p* ≤ 0.001; Fig. 6d, e, f). For both scenarios (all roots and roots by order class), R^2^ values exceeded 0.93 except for root length by root order, which was slightly lower (0.70). Digital reconstructions of the same root system (T4) using LMFD and TLS are presented in Fig. 3.

**Fig. 7:**
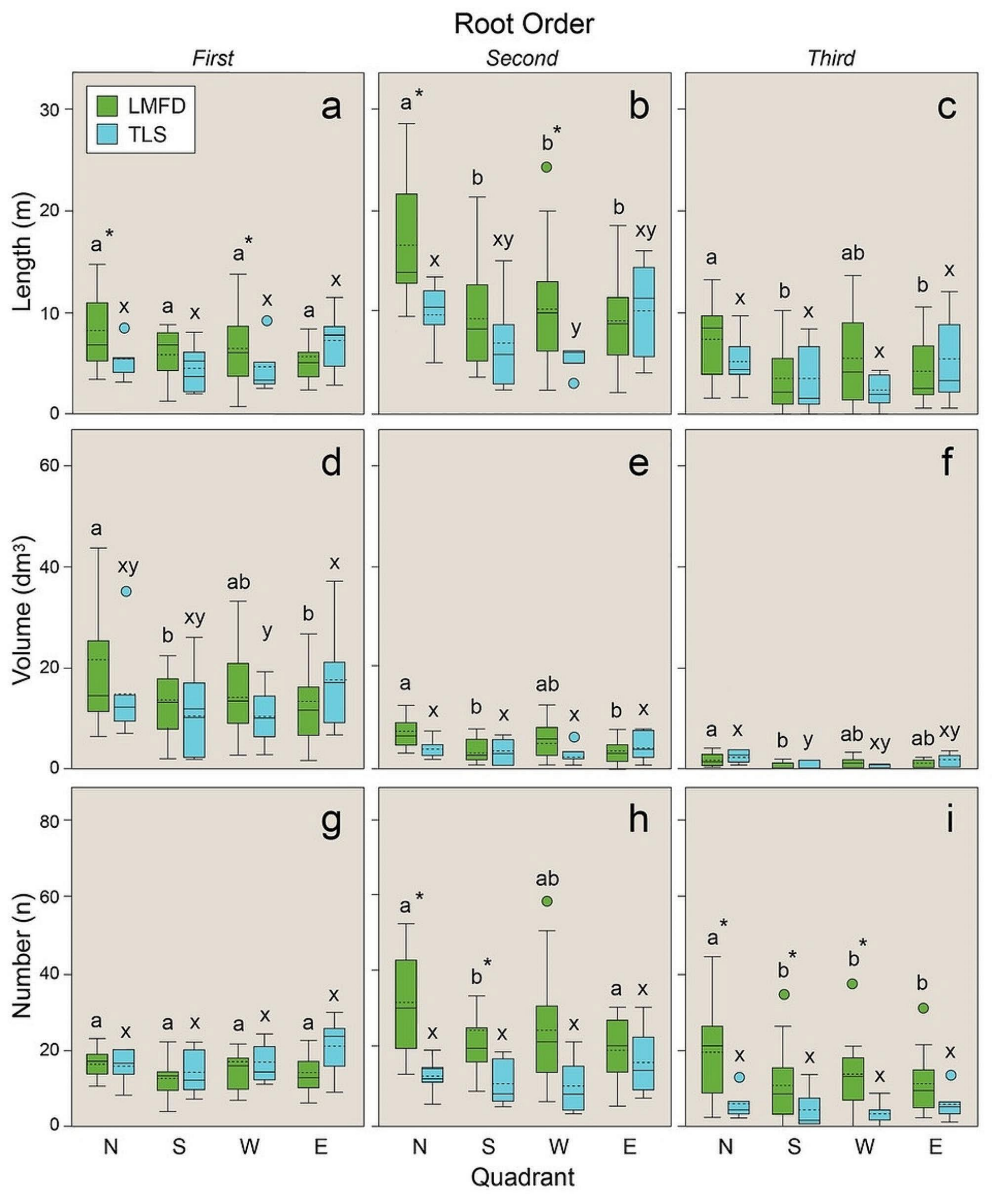
Length (m), volume (cm^3^), and number of first-, second-, and third-order roots by quadrant (N, north, downslope; S, south, upslope; W, west, windward; E, east, leeward) calculated according to LMFD (green) and TLS (blue). Letters within each root order and trait indicate significant differences (*p* < 0.05): a and b for differences within LMFD across quadrants, and x and y for differences within TLS across quadrants. Asterisks indicate a significant difference (*p* < 0.05) between the two methods within a quadrant. Vertical boxes represent approximately 50% of the observations, and lines extending from each box are the upper and lower 25% of the distribution. Circles represent outliers. Within each box, the solid horizontal line is the median and the dotted line is the mean

For the four cardinal direction sectors, the LMFD method yielded a significantly higher length than TLS for the first- and second-order roots in the downslope and windward quadrants (Fig. 7a, b). In the case of volume, no differences were detected among the two methods for the three root orders and the four directions analyzed (Fig. 7d, e, f). Finally, the number of roots did not differ between the two methods for the first-order roots only and in all the quadrants (Fig. 7g). The number of second- and third-order roots was significantly higher in LMFD than TLS for the windward and down- and up-slope quadrants (Fig. 7h, i).

## Discussion

Obtaining similar and consistent results among LMFD and TLS is important because errors in root trait measurement could lead to misinterpretation of the root system architecture and its asymmetrical spatial distribution. In fact, we have shown that the asymmetric distribution of roots in specific quadrants is strongly related to the response of a tree to the mechanical constraints (i.e., slope and wind) affecting tree anchorage [11, 12]. We observed, however, that reproduction of root system architecture with TLS was sometimes different than that with LMFD. Our result is not surprising given that LMFD requires in-hand assessment of roots.

Our comparison between LMFD and TLS showed optimal results of root traits such as length, volume, and number when analyzed as total roots. We observed, however, differences in these root traits when root order identification was considered, with TLS sometimes misidentifying orders by continuing along the wrong fork. Although significant, this inaccuracy was most pronounced with the measurement of length for first-order roots and for the volume of third-order roots that contributed in a smaller proportion respectively to the whole root system length than the more numerous and thinner higher-order root classes, and to the total root volume than the less numerous and thicker lower-order roots. Thus, while these differences between LMFD and TLS were statistically significant, they are less biologically significant since first-order roots do not influence the total length of a tree root system as higher root orders do [11].

When comparing root traits in the context of overall root system architecture (i.e., azimuthal distribution of roots within the four quadrants), we observed differences among the data collected by the two methodological approaches. On one hand, root length for first- and second-order roots, especially in the downslope (north) and windward (west) quadrants, differed significantly between methods. Trees generally produced more roots in these quadrants to dissipate mechanical forces associated with slope and wind [11]. On the other hand, values for root volume generally agreed between LMFS and TLS across the different root orders and quadrants. Regarding the number of first-order roots within each quadrant, we found similar values regardless of method, whereas we found that LMFD yielded significantly higher numbers of second and third-order roots within each quadrant, with the only exception of leeward (East). For TLS, our estimations of root trait totals (i.e., volume, length, and number) were dependent on the patch diameter used in the QSM, as evidenced by consistently strong trends across patch diameter classes. The estimates of these responses within each patch diameter class varied little, however, as evidenced by small standard deviations among the 12 models generated per class (results not shown).

Despite these observed differences, several evident advantages with TLS call for a continuation of the effort for its technological improvement. A potential advantage is that, in contrast to LMFD measurements that require the need to transport the excavated root systems from the field into the laboratory, TLS may be used in the field (in situ) without additional effort. In this study, we benefitted from the availability of LMFD data to evaluate the accuracy of TLS and our results suggest that if TLS were the only measurement system, then manual field measurement of a subsample of roots would be necessary. Marking selected roots with reflective tape would allow them to be identified in the subsequent TLS point cloud. Such roots could be hand-measured in the field and/or, after scanning, be destructively sampled and returned to the laboratory for oven drying to determine biomass/carbon measurements. These hand-measured values would improve the precision of the *PatchDiam* and QSM model parameterization, and hand-measured volumes could be divided by the volume of the whole root system as estimated from the TLS to calculate the proportion of root volume sampled. This proportion could then be applied to other attributes and/or traits to upscale the sampled root to the entire root system more accurately. This overall sampling approach is analogous to destructive subsampling of a tree crown to measure the biomass and volume of a subset of the branches for purposes of calibrating or validating an allometric model to estimate branch biomass and volume, based on TLS characterization of the crown volume.

Our observed discrepancies in root system architecture and the subsequent counts of the number of root numbers present in each spatial quadrant were likely caused by root occlusions given the fixed origin of each TLS scan; i.e., the three TLS scans per root system were designed to mitigate, but do not eliminate, occlusion effects. Additional in situ scans, especially from a higher view angle, may provide finer resolution leading to a more accurate identification of root classes and their branching point. Moreover, when we attempted to compare the reliability of the two methodologies in measuring root traits at two soil depths (< 30 cm and > 30 cm), we found poor alignment (see Fig. 3) and therefore omitted these results from this paper. With LMFD, the root system was suspended by supports placed under the first-order roots with the stump above (i.e., the natural position of a root system in the soil), whereas with TLS, we chose to invert the spatial orientation of the root systems (i.e., the stumps rested upside down on a pallet) to avoid vertical compression of the root system caused by the considerable weight of the stump. We believe that by doing so, we introduced an unnatural effect on the root system architecture, with long, thinner roots being affected by gravity unnaturally (e.g., they sagged appreciably). This sagging changed their relative depth position, causing a considerable effect on the analysis. Moreover, given that the stump cuts were not made consistently, neither perfectly horizontal nor consistently parallel with the slope, the resulting angles of inclination of all roots were different from their natural values. Thus, if roots are excavated and followed with *ex situ* TLS, we advise hanging the root systems right-side up to reproduce as closely as possible the three-dimensional position of the root system in the soil.

## Conclusions

In this first comparative test of root traits obtained either by Low Magnetic Field Digitization coupled with AMAPmod software or Terrestrial Laser Scanning coupled with TreeQSM software, we noted strong agreement for some traits and poor agreement for others. In general, at the total root system level, the two methods yielded comparable values for volume, length, and number. We therefore conclude that TLS could facilitate more rapid data collection in the field after excavation while retaining quantitative accuracy. TLS accuracy would likely be improved by scanning from at least two perspectives to reduce problems associated with occlusion, avoiding gravitational displacement as much as possible, and utilizing hand-measured subsamples to calibrate and validate QSM models, especially if more detailed measures by topology and spatial distribution criteria are desired. Despite some logistical challenges, our results suggest that future use of TLS has great promise for quantifying tree root system architecture in a rapid, replicable manner.

## Data Availability

Data reported here are available in the United States Department of Agriculture (USDA), Forest Service, Research Data Archive (Montagnoli et al. 2024).
