# Correction: Terrestrial laser scanning and low magnetic field digitization yield similar architectural coarse root traits for 32-year-old *Pinus ponderosa* trees

**DOI:** 10.1186/s13007-024-01245-9

**Published:** 2024-07-31

**Authors:** Antonio Montagnoli, Andrew T. Hudak, Pasi Raumonen, Bruno Lasserre, Mattia Terzaghi, Carlos A. Silva, Benjamin C. Bright, Lee A. Vierling, Bruna N. de Vasconcellos, Donato Chiatante, R. Kasten Dumroese

**Affiliations:** 1 Department of Biotechnology and Life Science, University of Insubria, Varese, Italy; 2 USDA Forest Service, Rocky Mountain Research Station, Moscow, ID USA; 3 Computing Sciences, Tampere University, Tampere, Finland; 4 Department of Biosciences and Territory, University of Molise, Pesche, Italy; 5 Department of Biosciences, Biotechnologies and Environment, University of Bari Aldo Moro, Bari, Italy; 6 School of Forest, Fisheries, and Geomatics Sciences, University of Florida, Gainesville, FL USA; 7 Department of Natural Resources and Society, University of Idaho, University Federal of Parana, Moscow, ID USA; 8 University Federal of Parana, Curitiba, Brazil

**Correction: Plant Methods (2024) 20: 102**

10.1186/s13007-024-01229-9

Following publication of the original article [1], the authors would like to correct the **Data availability** statement.

The sentence currently reads:

Data reported here are available in the United States Department of Agriculture (USDA), Forest Service, Research Data Archive (Montagnoli et al. 2021).

The correct sentence should be:

Data reported here are available in the United States Department of Agriculture (USDA), Forest Service, Research Data Archive (Montagnoli et al. 2024).

and the related reference:

Montagnoli A, Hudak AT, Raumonen P, de Vasconcellos BN, Bright BC, Silva CA, Vierling LA, Dumroese RK. Terrestrial laser scanning and low magnetic field digitization data for coarse roots of 32-year-old *Pinus ponderosa* trees. Fort Collins CO: USDA Forest Service Res Data Archive. 2024. 10.2737/RDS-2024-0050.

The original article has been corrected.

